# Risk factors for hospital readmission in chronic obstructive pulmonary disease: a systematic review and meta-analysis

**DOI:** 10.3389/fmed.2026.1836031

**Published:** 2026-07-14

**Authors:** Hao Tian, Wei Shen, Liuyang Huang, Bowen Feng, Baoyue Hong, Xianzheng Li, Fanrong Liang, Chunyan Yang, Qingguang Qin

**Affiliations:** 1 Department of Acupuncture, Hainan General Hospital, Hainan Affiliated Hospital of Hainan Medical University, Haikou, Hainan, China; 2 Hainan Medical University, Haikou, Hainan, China; 3 College of Acupuncture and Orthopedics, Hubei University of Chinese Medicine; Hubei Provincial Collaborative Innovation Center of Preventive Treatment by Acupuncture and Moxibustion; Hubei Shizhen Laboratory, Wuhan, Hubei, China; 4 Department of Rehabilitation, Hainan Provincial Hospital of Traditional Chinese Medicine, Haikou, Hainan, China; 5 College of Acupuncture and Tuina, The Third Teaching Hospital, College of Basic Medicine, College of International Education, Chengdu University of Traditional Chinese Medicine; Clinical Research Center for Acupuncture and Moxibustion in Sichuan Province, Chengdu, Sichuan, China; 6 Department of Acupuncture and Massage, Shenzhen Second People’s Hospital, The First Affiliated Hospital of Shenzhen University, Shenzhen, China

**Keywords:** chronic obstructive pulmonary disease, hospital readmission, readmission rate, risk factors, systematic review, meta-analysis

## Abstract

**Background:**

Patients with chronic obstructive pulmonary disease (COPD) commonly experience acute exacerbations that require hospitalization and may lead to repeated COPD-related readmissions. Among patients who survive an index hospitalization, early readmission due to acute exacerbation of COPD remains a major clinical challenge. Therefore, evaluating readmission rates at different follow-up time points and identifying factors associated with readmission are important for improving COPD management. Better risk assessment and early recognition of high-risk patients may support targeted interventions, reduce avoidable readmissions, and improve quality of life and clinical outcomes.

**Objective:**

This study aimed to systematically summarize COPD-related readmission rates within 1 month, 3 months, 6 months, and 1 year after hospital discharge, and to identify factors associated with COPD-related readmission.

**Methods:**

Eight electronic databases were searched from inception to October 1, 2026, to identify observational studies reporting COPD-related readmission. Methodological quality was assessed using the Newcastle–Ottawa Scale (NOS) for cohort and case–control studies and an adapted NOS for cross-sectional studies. Pooled estimates of readmission rates and factors associated with readmission among patients with COPD were calculated using random-effects models.

**Results:**

A total of 54 studies were included, reporting 25 outcome indicators. The pooled readmission rates after discharge were 15.2% within 1 month, 26.90% within 3 months, 30.40% within 6 months, and 42.10% within 1 year. In total, 26 factors associated with readmission were identified. Overall, the methodological quality of the included studies was moderate to high.

**Conclusion:**

This meta-analysis examined readmission rates and factors associated with readmission among patients with COPD. The findings show that the 1-year readmission remains common and is influenced by multiple clinical factors. These results underscore the need for early identification of high-risk patients and timely interventions to improve long-term COPD management. Sensitivity analyses showed that the findings were stable, and the included studies were generally of moderate to high methodological quality, supporting the reliability and clinical relevance of the conclusions.

**Systematic review registration:**

https://www.crd.york.ac.uk/PROSPERO/view/CRD420261286213, Identifier CRD420261286213.

## Introduction

Chronic obstructive pulmonary disease (COPD) is characterized by persistent airflow limitation and chronic airway inflammation. It is highly prevalent among older adults and remains a leading cause of morbidity and mortality worldwide, placing a substantial socioeconomic burden on healthcare systems (1–3). Unplanned readmission refers to an unexpected rehospitalization for the same or a related condition after discharge from a previous hospital stay (4). Patients with COPD are particularly vulnerable to acute exacerbations, which often require hospitalization and may lead to repeated readmissions. Among those who survive an index hospitalization, early readmission after discharge remains a major clinical challenge (5). Readmission after hospitalization for COPD is common, especially in Asian populations, where 1-month readmission rates have been reported to range from 11.0 to 25.5% (6–8). Acute exacerbations occur approximately 0.5 to 3.5 times per year, and hospitalization accounts for a substantial proportion of the annual direct medical costs of COPD. Nearly 20% of patients hospitalized for acute exacerbations are readmitted within 30 days, resulting in considerable healthcare expenditure (9). In addition, the Hospital Readmissions Reduction Program imposes financial penalties on hospitals when readmissions for acute exacerbations of COPD exceed expected levels (10). Frequent exacerbations accelerate disease progression, worsen lung function, reduce quality of life, and increase the risks of COPD-related readmission and mortality, thereby imposing a heavy burden on patients, families, and healthcare systems (11–13).

The 2023 Global Initiative for Chronic Obstructive Lung Disease (GOLD) guidelines emphasize that clinical assessment of COPD should not be based solely on lung function parameters, such as predicted FEV₁%. Instead, GOLD recommends a multidimensional approach that considers both the frequency and severity of acute exacerbations and patient-reported symptoms, including the COPD Assessment Test and the modified Medical Research Council dyspnea scale. In particular, exacerbation history over the preceding 12 months is a key component of risk stratification. According to GOLD 2023, patients who have two or more acute exacerbations within 1 year, or at least one exacerbation requiring hospitalization, are classified as high risk and should receive management strategies tailored to this population (14). This recommendation highlights the central role of hospitalization and readmission events in COPD risk assessment and long-term disease management. Readmissions not only indicate disease instability and suboptimal treatment response but are also closely linked to worsening overall health. Frequent exacerbations accelerate lung function decline, impair quality of life, limit daily activities, and increase psychological burden (15). Previous studies have reported that 1-year rehospitalization rates after an initial hospitalization may reach 30–40%, and that readmission is an independent predictor of increased mortality risk. Readmissions are also associated with poorer quality of life, greater healthcare use, and higher economic burden (16). Therefore, in both clinical practice and interventional research, readmission is a clinically meaningful outcome for evaluating COPD prognosis and management.

However, despite increasing attention to readmission, substantial differences remain across studies in reported readmission rates, follow-up periods, study populations, healthcare settings, and regions. Although factors such as disease severity, comorbidities, and previous hospitalization are commonly associated with readmission, their effect sizes and relative contributions vary across studies (6, 17). In addition, pooled estimates of readmission rates at different time points after discharge have not been comprehensively summarized. Given the strong association of readmission with disease progression, mortality, healthcare use, and economic burden, a clearer understanding of readmission patterns and related factors is essential for improving risk stratification and post-discharge management in patients with COPD. Therefore, this systematic review and meta-analysis aimed to estimate pooled readmission rates at different post-discharge time points and identify factors associated with COPD-related readmission, providing evidence to support targeted prevention strategies and optimized clinical care (18).

## Methods

### Search strategy

We systematically searched the VIP Database, China National Knowledge Infrastructure, Chinese Biomedical Literature Database, WanFang Database, PubMed, Web of Science, Cochrane Central Register of Controlled Trials, and Embase to identify studies reporting readmission rates and factors associated with readmission among patients with COPD. Searches were conducted from database inception to October 1, 2026. Search strategies combined subject headings and free-text terms. English search terms included terms related to COPD, such as “chronic obstructive pulmonary disease,” “chronic obstructive lung disease,” “chronic obstructive airway disease,” “chronic airflow obstruction,” and “COPD”; terms related to readmission, such as “readmission,” “rehospitalization,” “re-hospitalization,” and “multiple admission”; and terms related to potential determinants, such as “associated factors,” “predictor,” “recurrence,” “protective factor,” “contributing factor,” and “predictive.” This systematic review and meta-analysis was registered in PROSPERO (CRD420261286213). Representative full search strategies for both English and Chinese databases are provided in Supplementary material 2.

### Inclusion and exclusion criteria

Studies were included if they met the following criteria: (1) Patients were diagnosed with COPD according to the GOLD guidelines (19) or the Chinese Guidelines for the Diagnosis and Treatment of Chronic Obstructive Pulmonary Disease (20); (2) The study reported COPD-related readmission within 1 year after discharge as a binary outcome; (3) Readmission rates and factors associated with COPD-related readmission were reported, with multivariable-adjusted odds ratios (ORs) and corresponding 95% confidence intervals (CIs) available or extractable from the original study; (4) The study design was cross-sectional, cohort, or case–control; and (5) Patients were grouped according to different exposure characteristics or readmission status.

### Exclusion criteria

Studies were excluded if they met any of the following criteria: (1) duplicate publications based on the same study population, in which case only the study with the most comprehensive data was retained; (2) insufficient or unverifiable data for estimating readmission rates or extracting adjusted odds ratios; (3) conference abstracts or proceedings (4) full text unavailable; and (5) NOS score <5 (21).

### Data selection

Study selection was conducted in two stages. First, duplicate records were removed using EndNote software (Version No.: Bld 18,631; Thomson Reuters, New York, USA). Titles and abstracts were then screened to exclude studies that clearly did not meet the eligibility criteria. Second, two reviewers independently assessed the full texts of potentially eligible studies according to the predefined inclusion and exclusion criteria. Any disagreements were resolved through discussion or consultation with a third reviewer. Studies with incomplete or unverifiable data were excluded. Data were extracted using a standardized data extraction form. Extracted information included first author, year of publication, patient sex, data source, study design, country, sample size, COPD-related readmission rate, factors associated with COPD-related readmission, comorbidities, and outcome measures. The main outcomes were COPD-related readmission rates and factors associated with COPD-related readmission.

### Quality assessment

Literature quality was assessed according to the NOS criteria (22, 23). The assessment covered the following domains: (1) adequacy of case definition; (2) representativeness of cases; (3) adequacy of control definition; (4) representativeness of controls; (5) comparability of cases and controls; (6) ascertainment of exposure; (7) comparability of exposure ascertainment between cases and controls; and (8) non-response rate. Each item was assigned one point, with a maximum total score of nine. Studies with a score of ≥6 were considered high quality. Quality assessment was performed independently by two reviewers, and the results were cross-checked. Disagreements were resolved through discussion; when consensus could not be reached, a third reviewer was consulted for final adjudication.

### Statistical analysis

This meta-analysis was performed using R software version 4.4.2 with the meta package. COPD-related readmission rates and corresponding 95% confidence intervals (CIs) were used to estimate pooled readmission rates, while adjusted odds ratios (ORs) and corresponding 95% CIs were used to assess factors associated with COPD-related readmission (24). Given the anticipated clinical and methodological heterogeneity across the included observational studies, including differences in study design, COPD severity, study populations, healthcare settings, follow-up durations, definitions of COPD-related readmission, and variables associated with readmission, random-effects models were applied *a priori* for all meta-analyses. Statistical heterogeneity was assessed using the χ^2^ test and quantified with the *I^2^* statistic (25). Sensitivity analyses were conducted to assess the robustness of the pooled results when substantial heterogeneity was observed.

### Risk of bias assessment

Publication bias was assessed quantitatively using Begg’s and Egger’s tests (26). Statistical significance was defined as *p* < 0.10; therefore, a *p* value ≥0.10 was interpreted as indicating no evidence of significant publication bias (27).

### Sensitivity analysis

To assess the robustness of the findings, sensitivity analyses were performed by sequentially excluding one study at a time and recalculating the pooled estimates. The influence of each study was evaluated by comparing the recalculated estimates with the overall pooled estimate. Results were considered stable if the exclusion of any single study did not substantially change the pooled estimate, whereas marked changes indicated greater sensitivity and reduced robustness. Because only three studies reported 6-month readmission rates, this outcome was examined descriptively to explore potential sources of heterogeneity.

## Results

### Study search and selection

**Figure 1:**
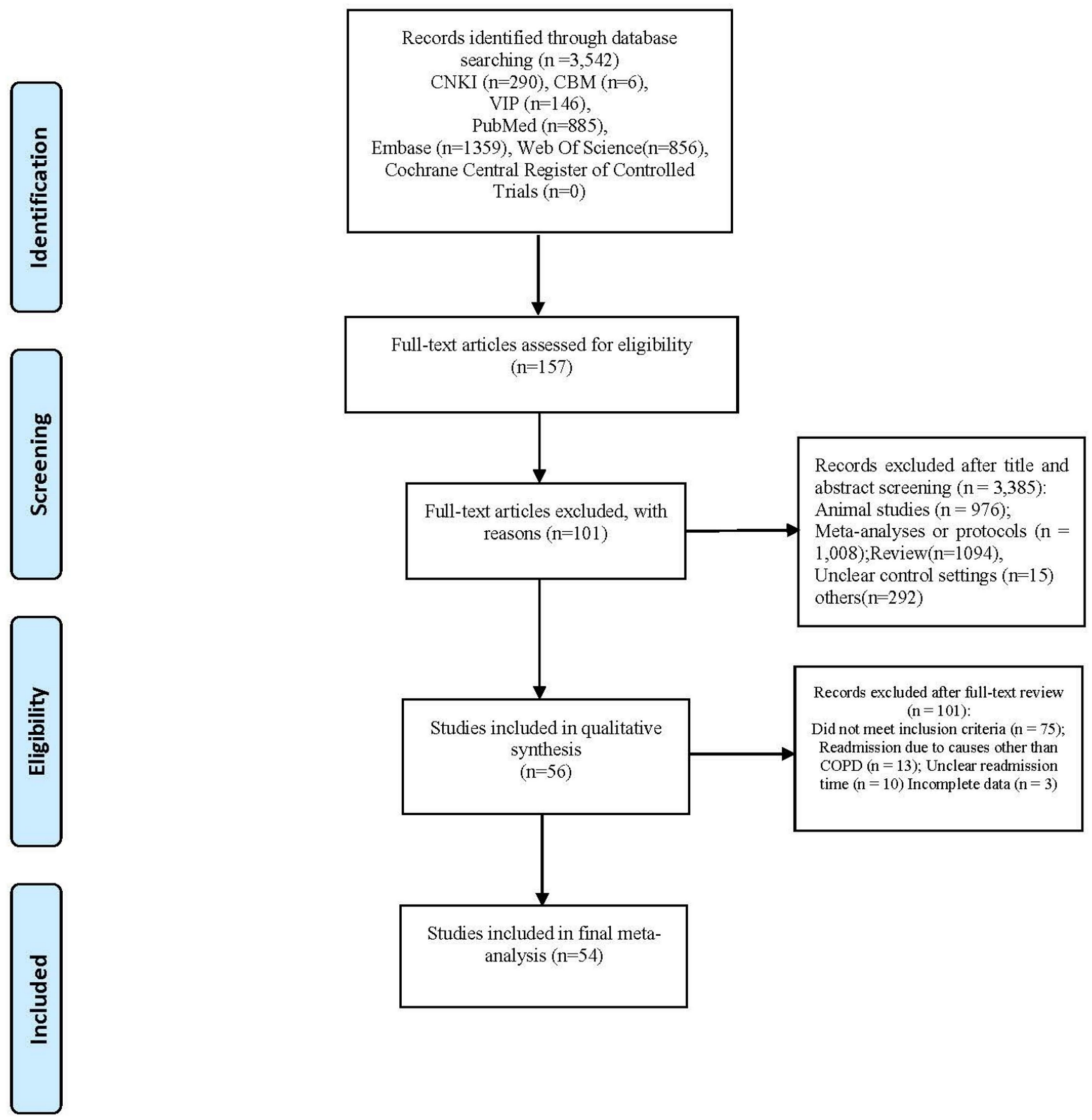
Flow diagram of study selection.

A total of 3,542 records were identified through database searches. After title and abstract screening, 3,385 records were excluded, including animal studies, meta-analyses or protocols, reviews, studies with unclear control or outcome settings, and other irrelevant records. The remaining 157 articles underwent full-text assessment. Of these, 101 articles were excluded because they did not meet the inclusion criteria, reported readmissions due to causes other than COPD, had unclear readmission time points, or provided incomplete or unverifiable data. Finally, 56 studies were included in the qualitative synthesis, of which 54 were included in the final meta-analysis. The study selection process is shown in Figure 1.

### Characteristics of included studies

**Figure 2:**
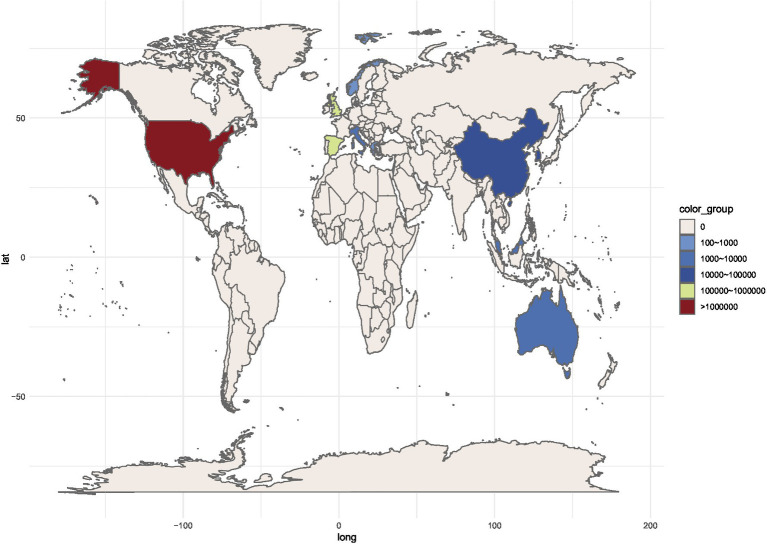
Geographic distribution of the included studies and participant sample sizes. Countries are colored according to the total number of participants contributed by studies conducted in each country.

A total of 54 studies were ultimately included, comprising 31,161,712 participants. Of these, 29 were retrospective studies (18, 28–55), 21 were prospective studies (56–76), and 4 were case control studies (77–80). All studies were published between 2004 and 2024. Regarding geographic distribution, 25 studies were conducted in China (18, 42–44, 46, 50, 52–54, 56, 57, 62, 66–74, 76, 78–80), 10 in the United States (31, 33, 38–41, 47, 48, 59, 75),five in the United Kingdom (28, 36, 51, 58, 61), seven in Spain (29, 30, 34, 37, 45, 49, 55), and one study each in Singapore (64), Norway (77), Australia (63), Greece (60), Malaysia (65), Italy (35) and Israel (32) (Figure 2). The mean age of participants ranged from 35 to over 85 years across the included studies, and reported readmission rates ranged from 3.5 to 60.59%. Factors associated with readmission included sex, age, race or ethnicity, payment method, discharge destination, comorbidity burden, arterial oxygen level, alcohol consumption, smoking history, prior COPD exacerbations, number of hospitalizations in the previous year, previous emergency department visits, length of hospital stay, lung function, dyspnea severity, mMRC score, use of ventilatory support, pharmacological treatment, long-term oxygen therapy, malnutrition, BMI, BNP level, inflammatory markers, anxiety, depression, and comorbid conditions such as cardiovascular disease, anemia, osteoporosis, and metabolic disorders. The duration of follow-up varied across studies, ranging from 3 to 365 days. The most commonly reported time points were 1 month (31 ± 3 days), 3 months (91 ± 3 days), 6 months (182 ± 3 days), and 1 year (363 ± 3 days). All included studies had NOS scores ≥6, indicating overall moderate to high methodological quality (Supplementary Table 1).

### Qualitative synthesis of included studies

The included studies identified a broad range of factors associated with readmission, mainly covering demographic characteristics, disease severity, comorbidity burden, nutritional status, psychological factors, inflammatory markers (31, 34, 43, 53, 54, 72, 76, 78–80), healthcare utilization, and respiratory support. The most frequently reported factors associated with increased readmission risk were sex (28, 31, 35, 40, 45, 47–52, 55, 56, 63, 73, 75), higher Charlson Comorbidity Index (CCI) scores (32, 45, 47, 49, 52, 55, 56, 58, 59, 63), impaired lung function (28, 43, 46, 73), hypoxemia (34, 43, 50, 53, 57, 60), previous hospitalization (29, 32, 33, 37, 40, 42, 43, 46, 48, 50, 52, 55, 60, 62, 70–72, 74), malnutrition (45, 47, 49, 71, 74), depression (36, 39, 60, 69), and prior use of ventilatory support (45, 46, 58, 60).

Several studies showed that patients with more severe disease, reflected by lower FEV₁%, more severe dyspnea, previous acute exacerbations, and chronic hypoxemia, were more likely to be readmitted after discharge. Comorbidity burden was also frequently reported, particularly in patients with cardiovascular disease, anemia, osteoporosis, or metabolic disorders. Psychological and nutritional factors, including depression and malnutrition, were also associated with readmission. In addition, indicators of prior healthcare use and clinical instability, such as previous emergency department visits, longer hospital stays, and prior use of ventilatory support, were commonly linked to higher readmission rates.

However, findings were inconsistent for several variables, including age, smoking history, inflammatory markers, and some chronic comorbidities. This inconsistency may be partly explained by differences in follow-up duration, healthcare setting, study population, disease severity, and definitions of readmission. Therefore, variables reported in at least two studies with comparable definitions were included in the quantitative meta-analysis.

### Quantitative synthesis

#### Readmission rates

**Figure 3:**
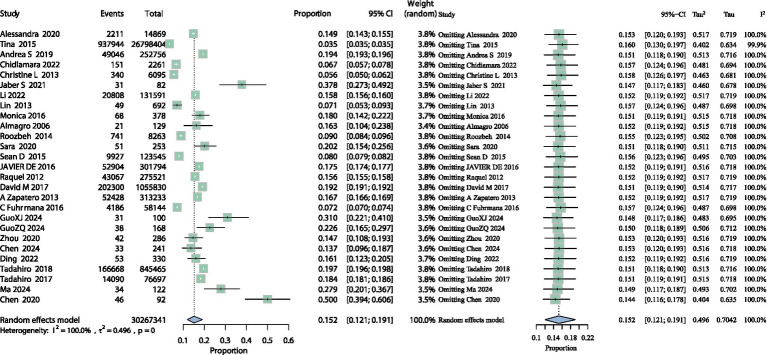
Forest plot of 1-month COPD readmission rates.

**Figure 4:**
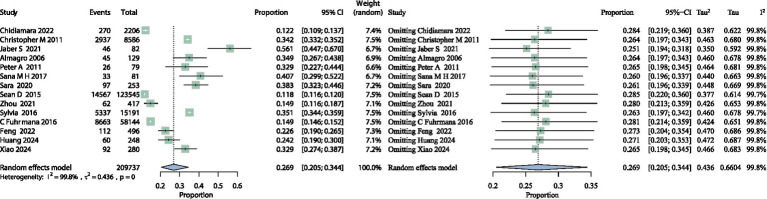
Forest plot of 3-month COPD readmission rates.

**Figure 5:**
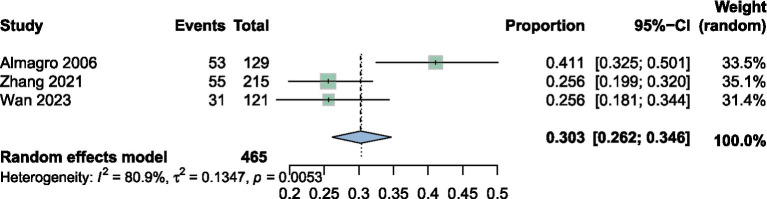
Forest plot of 6-month COPD readmission rates.

**Figure 6:**
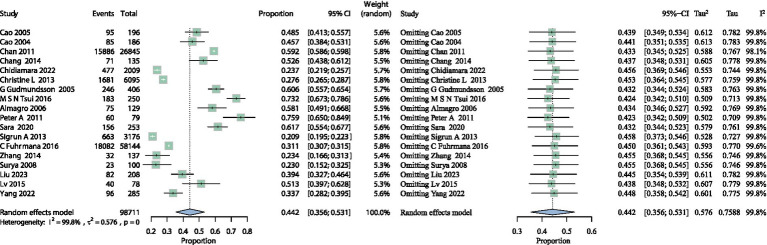
Forest plot of 1-year COPD readmission rates.

Readmission rates varied substantially across studies. Among patients with COPD, 1 month readmission rates ranged from 3.50 to 50.00% across 27 studies (18, 30, 33, 35–41, 45, 47–49, 51–55, 57, 59, 60, 63, 72, 74, 76, 79). The corresponding ranges were 11.79 to 56.10% for 3-month readmission in 14 studies (28, 30, 37, 41, 48, 58, 59, 61, 63, 65, 66, 69, 71, 78), 25.60 to 41.10% for 6-month readmission in three studies (30, 42, 70), and 20.88 to 75.95% for 1-year readmission in 18 studies (28, 30, 31, 37, 40, 43, 44, 46, 48, 50, 55, 56, 63, 64, 67, 73, 75, 77). The pooled readmission rates were 15.20% within 1 month (95% CI: 12.30–18.50%; *I^2^* = 100.00%), 26.90% within 3 months (95% CI: 20.50–34.40%; *I^2^* = 99.80%), 30.40% within 6 months (95% CI: 21.50–40.90%; *I^2^* = 80.90%), and 42.10% within 1 year (95% CI: 33.60–51.10%; *I^2^* = 98.90%). Sensitivity analyses performed by sequentially excluding individual studies identified no influential outliers for the 3-month or 1-year readmission estimates, supporting the robustness of these pooled results. Only three studies were available for the 6-month readmission analysis. Although all three studies used a 6-month follow-up window, Zhang et al. (70) had a substantially larger sample size than the other two studies and may therefore have contributed greater weight to the pooled estimate. As a result, the pooled 6-month estimate may be more sensitive to this study. Differences in sample characteristics, geographic settings, and admission criteria may also have contributed to the observed heterogeneity in this analysis (*I^2^* = 80%) (Figures 3–6).

### Factors associated with 1-month readmission

A comprehensive meta-analysis was conducted to identify factors associated with readmission within one month among patients with COPD. Regarding sex, nine studies involving male patients and five studies involving female patients demonstrated substantial heterogeneity (*I^2^* = 99.4%, *p* = 0.003; *I^2^* = 99.0%, *p* < 0.001). The pooled results showed that male sex was positively associated with one month readmission (OR = 1.252, 95% CI: 1.081–1.451, *p* < 0.001), whereas female sex showed a modest inverse association (OR = 0.931, 95% CI: 0.875–0.991, *p* = 0.026). For oxygenation status, two studies evaluated PaO₂ ≤ 60 mmHg, with no heterogeneity observed (*I^2^* = 0%, *p* = 0.561). PaO₂ ≤ 60 mmHg was associated with higher odds of 1-month readmission (OR = 1.022, 95% CI: 1.009–1.035, *p* < 0.001), whereas a higher PaO₂/FiO₂ ratio was inversely associated with readmission (OR = 0.980, 95% CI: 0.970–0.990, *p* < 0.001). For laboratory indicators, CRP (OR = 1.846, 95% CI: 1.497–2.276, *p* < 0.001) and RDW (OR = 2.070, 95% CI: 1.320–3.246, *p* = 0.002) were associated with higher odds of 1-month readmission. Studies evaluating CRP (*I^2^* = 28.8%, *p* = 0.229) and RDW (*I^2^* = 0%, *p* = 0.492) showed low heterogeneity. Regarding comorbidity burden, studies assessing CCI scores of 1 (3 studies), 2 (8 studies), and ≥3 (5 studies) all exhibited substantial heterogeneity (*I^2^* > 96%). The pooled analysis showed that CCI scores of 2 (OR = 1.154, 95% CI: 1.053–1.265, *p* = 0.002), ≥3 (OR = 1.522, 95% CI: 1.491–1.553, *p* < 0.001), and ≥5 (OR = 2.250, 95% CI: 1.663–3.045, *p* < 0.001) were significantly associated with higher odds of 1-month readmission. Prior ventilatory support was also associated with higher odds of 1-month readmission (OR = 1.274, 95% CI: 1.046–1.551, *p* = 0.016), although substantial heterogeneity was observed across the four studies (*I^2^* = 94.9%, *p* < 0.001). Among chronic comorbidities, anemia (OR = 1.250, 95% CI: 1.213–1.288, *p* < 0.001) and osteoporosis (OR = 1.179, 95% CI: 1.072–1.296, *p* = 0.005) were associated with higher odds of 1-month readmission, whereas obesity showed an inverse association (OR = 0.850, 95% CI: 0.812–0.890, *p* < 0.001). A history of alcohol use was also associated with higher odds of 1-month readmission (OR = 1.129, 95% CI: 1.095–1.164, *p* = 0.016), with no heterogeneity observed across the two studies (*I^2^* = 0%, *p* = 0.777). For length of hospital stay, studies assessing ≤7 days (8 studies) and >7 days (10 studies) both showed substantial heterogeneity (*I^2^* > 99%). Both hospital stays of ≤7 days (OR = 1.189, 95% CI: 1.087–1.301, *p* < 0.001) and >7 days (OR = 1.442, 95% CI: 1.099–1.893, *p* = 0.008) were associated with higher odds of 1-month readmission. Malnutrition was also associated with higher odds of 1-month readmission (OR = 1.438, 95% CI: 1.204–1.717, *p* < 0.001), although substantial heterogeneity was observed across the five studies (*I^2^* = 92.1%, *p* < 0.001). Previous emergency department visits were associated with higher odds of 1-month readmission (OR = 1.265, 95% CI: 1.060–1.510, *p* = 0.009), with high heterogeneity across the eight studies (I^2^ = 99.3%, *p* < 0.001). Comorbid depression was also associated with higher odds of 1-month readmission (OR = 1.096, 95% CI: 1.041–1.153, *p* < 0.001), with no heterogeneity observed across the two studies (*I^2^* = 0%, *p* = 0.631).

In contrast, WBC, NLR, and BNP were not significantly associated with 1-month readmission. A CCI score of 1 was also not statistically significant. Other chronic comorbidities were not significantly associated with readmission. In addition, age categories of 45–65, 66–75, 75–85, and ≥85 years, history of acute exacerbations in the previous year, FEV₁% < 50, use of COPD-related medications, and long-term oxygen therapy (LTOT) in the previous year were not significantly associated with 1-month readmission (Supplementary Table 2).

### Factors associated with readmission within 3-months

11 studies reported estimates for male sex and five for female sex. Both analyses showed substantial heterogeneity (male: *I^2^* = 99.4%, *p* = 0.003; female: *I^2^* = 99.0%, *p* < 0.001). Male sex was associated with higher odds of 3-month readmission in patients with COPD (OR = 1.252, 95% CI: 1.081–1.451, *p* < 0.001), whereas female sex was associated with lower odds of readmission (OR = 0.931, 95% CI: 0.875–0.991, *p* = 0.026).

For age, studies evaluating patients aged 66–75 years showed high heterogeneity. This age group was associated with lower odds of 3-month readmission (OR = 0.269, 95% CI: 0.205–0.344, *p* = 0.015). Regarding payment method, six studies evaluating public medical insurance or assistance, including national and employee medical insurance, showed substantial heterogeneity (*I^2^* = 99.6%, *p* < 0.001). The pooled result indicated that this payment category was associated with increased odds of 3-month readmission (OR = 1.158, 95% CI: 1.029–1.303, *p* = 0.015). Comorbidity burden was also associated with 3-month readmission. Two studies assessing patients with a CCI of 2 showed no heterogeneity (*I^2^* = 0%, *p* = 0.463). Both CCI = 2 (OR = 1.105, 95% CI: 1.041–1.173, *p* = 0.001) and CCI ≥ 3 (OR = 1.300, 95% CI: 1.207–1.401, *p* < 0.001) were associated with higher odds of readmission within 3 months. For ventilatory support, two studies were included, and prior receipt of ventilatory support was associated with increased odds of 3-month readmission among patients with COPD (OR = 1.130, 95% CI: 1.035–1.233, *p* = 0.006).

In contrast, no statistically significant associations were observed for private insurance or self-payment. A CCI score of 1, PaO₂ ≤ 60 mmHg, FEV₁% < 50, and chronic comorbidities, including diabetes mellitus, ischemic heart disease, stroke, and cancer, were also not significantly associated with readmission within 3 months (Supplementary Table 3).

### Factors associated with 6-month readmission

Two studies evaluated BMI < 18.5 kg/m^2^, and no heterogeneity was observed (*I^2^* = 0%, *p* = 0.817). The pooled result showed that BMI < 18.5 kg/m^2^ was associated with higher odds of readmission within 6-months (OR = 5.329, 95% CI: 2.552–11.128, *p* < 0.001). For acute exacerbations in the previous year, two studies reported data for patients with ≥2 exacerbations, with no heterogeneity observed (*I^2^* = 0%, *p* = 0.997). The pooled analysis showed that having ≥2 acute exacerbations in the previous year was associated with higher odds of 6-month readmission (OR = 3.004, 95% CI: 1.599–5.642, *p* < 0.001).

### Factors associated with 1-year readmission

Seven studies assessed impaired lung function defined as FEV₁% < 50, with substantial heterogeneity observed (*I^2^* = 75.2%, *p* < 0.001). The pooled result showed that FEV₁% < 50 was associated with higher odds of 1-year readmission among patients with COPD (OR = 1.364, 95% CI: 1.026–1.814, *p* = 0.033). Four studies evaluated comorbid depression, with no significant heterogeneity observed (*I^2^* = 0%, *p* = 0.603). Comorbid depression was associated with increased odds of 1-year COPD-related readmission (OR = 1.370, 95% CI: 1.139–1.649, *p* < 0.001). Four studies assessed BMI < 18.5 kg/m^2^, with moderate heterogeneity (*I^2^* = 63.4%, *p* = 0.042). The pooled analysis showed that low BMI was associated with higher odds of 1-year readmission (OR = 3.014, 95% CI: 1.692–5.370, *p* < 0.001). For dyspnea severity, four studies evaluated severe dyspnea, including higher mMRC scores, with low heterogeneity (*I^2^* = 41.0%, *p* = 0.166). Severe dyspnea was significantly associated with higher odds of 1-year readmission among patients with COPD (OR = 2.021, 95% CI: 1.498–2.726, *p* < 0.001) (Supplementary Table 4).

### Publication bias

**Figure 7:**
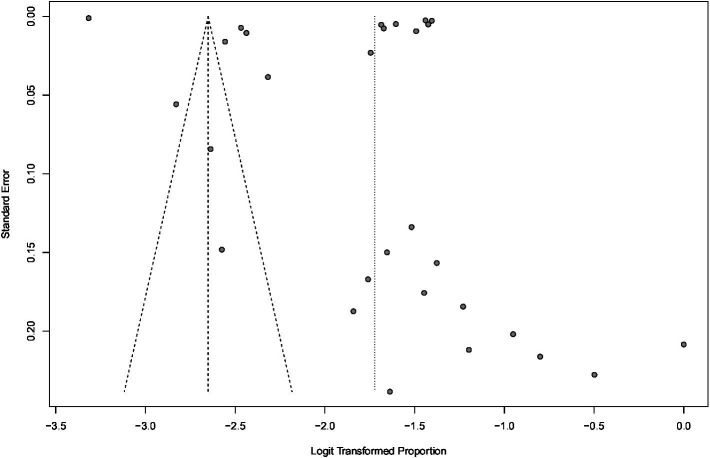
Funnel plot of one month COPD readmission rates.

**Figure 8:**
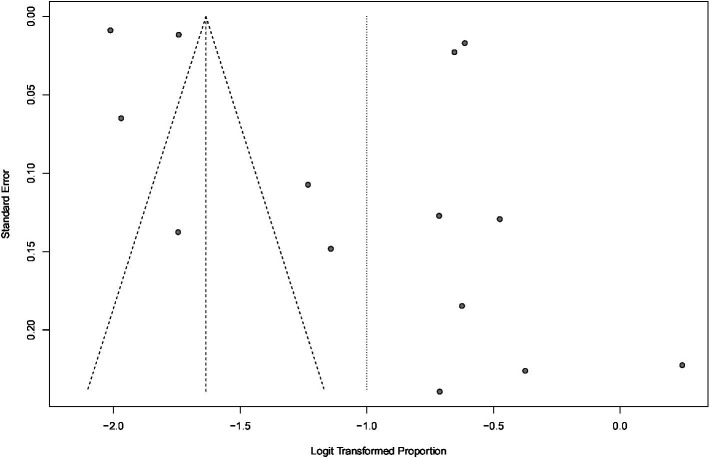
Funnel plot of three-month COPD readmission rates.

**Figure 9:**
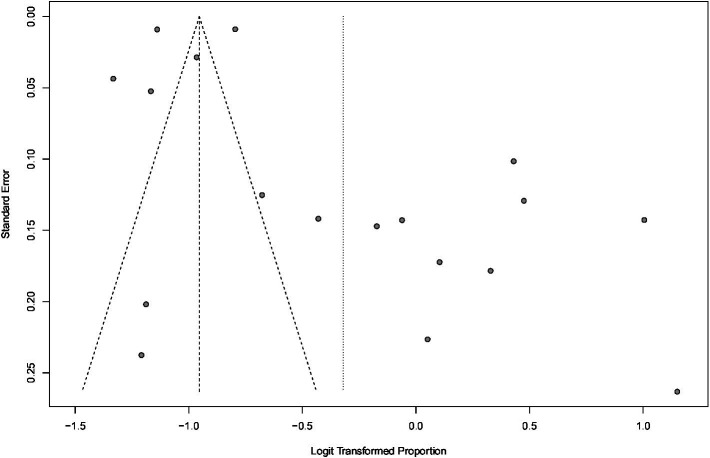
Funnel plot of one year COPD readmission rates.

Publication bias was assessed using funnel plots for readmission rates within 1 month, 3 months, and 1 year. Overall, the funnel plots showed varying degrees of asymmetry across the three time points. For 1-month readmission, both Begg’s test (*p* < 0.001) and Egger’s test (*p* = 0.048) suggested potential publication bias. For 3-month readmission, neither Begg’s test (*p* = 0.412) nor Egger’s test (*p* = 0.212) indicated significant publication bias. For 1-year readmission, Begg’s test did not suggest publication bias (*p* = 0.519), whereas Egger’s test indicated possible small-study effects or publication bias (*p* = 0.061). The funnel plots are shown in Figures 7–9

## Discussion

The present systematic review and meta-analysis aimed to quantify readmission rates among patients with COPD at different time points after discharge and to identify factors associated with readmission risk. The pooled readmission rates increased over time, reaching 15.2% within 1 month, 26.9% within 3 months, 30.4% within 6 months, and 42.1% within 1 year after discharge. These findings indicate that readmission is not confined to the early transition period after hospitalization but remains a persistent and clinically important burden throughout the first post-discharge year. In particular, the high 1-year readmission rate highlights the long-term vulnerability of patients with COPD and underscores the need for sustained follow-up and post-discharge management. The pooled readmission rates observed in this study were generally consistent with previous reports, although some differences were noted. The 1-year readmission rate of 42.1% was slightly higher than that reported in earlier meta-analyses (6). This discrepancy may be explained by differences in study populations, healthcare systems, COPD severity, follow-up duration, and definitions of readmission across studies. The progressive increase in readmission rates over time further suggests that post-discharge COPD management should not focus only on short-term prevention. Instead, care should extend across the first year after hospitalization. Early transitional care may be particularly important for preventing short-term readmissions, whereas long-term disease control, comorbidity management, and pulmonary rehabilitation may be more relevant for reducing later rehospitalization.

The qualitative synthesis further showed that COPD readmission is a multifactorial outcome. It is influenced by demographic characteristics, disease severity, comorbidity burden, nutritional status, psychological status, healthcare use, and respiratory support. Across the included studies, male sex, higher CCI scores, impaired lung function, severe dyspnea, malnutrition, depression, and previous ventilatory support were among the most consistently reported factors associated with increased readmission risk. However, substantial differences were observed across studies in patient populations, healthcare settings, follow-up durations, and definitions of readmission-related variables. These differences may partly explain the heterogeneity observed in the quantitative analyses.

For variables with sufficiently comparable definitions, the meta-analysis showed that COPD readmission is not driven by a single factor. Rather, it reflects the combined influence of patient characteristics, disease severity, comorbidity burden, and overall health status. Male sex was associated with a higher risk of readmission, which may partly reflect the historically higher prevalence of smoking among men. Smoking contributes to COPD onset and progression and may increase the risk of acute exacerbation and rehospitalization through persistent airway inflammation, oxidative stress, and accelerated lung function decline (81). However, some studies have reported more severe exacerbations and higher hospitalization rates among female patients with COPD (82), suggesting that the relationship between sex and COPD outcomes is complex. Future studies should therefore further examine sex-specific differences in COPD progression, exacerbation risk, and post-discharge outcomes. Greater comorbidity burden and more severe respiratory impairment were also associated with readmission risk. In this study, higher CCI, reduced FEV₁%, severe dyspnea, and previous ventilatory support were associated with a higher likelihood of rehospitalization. A higher comorbidity burden reflects multimorbidity and reduced physiological reserve, whereas hypoxemia, impaired lung function, and the need for ventilatory support indicate more advanced disease and persistent instability after discharge. These findings suggest that patients with substantial systemic burden and residual respiratory dysfunction are particularly vulnerable to recurrent clinical deterioration. Previous studies have similarly shown that comorbidity burden is associated with increased risks of readmission and mortality among patients with COPD (83). These high-risk patients may therefore benefit from comprehensive pre-discharge assessment, optimized management of comorbid conditions, and closer post-discharge follow-up, including reassessment of the need for long-term oxygen therapy (LTOT) and noninvasive ventilation (NIV) when clinically appropriate (84). Psychological and nutritional status also represent important dimensions of readmission risk. In this study, depression and malnutrition were both associated with increased risk of rehospitalization. Depression may reduce treatment adherence, self-management capacity, and social functioning, thereby increasing the likelihood of recurrent hospitalization. Malnutrition may contribute to skeletal muscle wasting, impaired respiratory muscle function, and reduced immune competence, increasing susceptibility to infection, acute exacerbations, and readmission (47, 85). These findings suggest that readmission risk assessment should not be limited to traditional respiratory indicators, but should also include psychological screening and nutritional evaluation as part of discharge planning and follow-up care. Taken together, these findings support a multidimensional and risk-stratified approach to reducing COPD readmissions. Given the substantial burden of readmission during the first year after discharge, clinical care should include both early transitional support and sustained long-term management. Potential strategies include structured discharge planning, patient education, inhaler technique training, individualized action plans for exacerbations, smoking cessation support, nutritional intervention, pulmonary rehabilitation, and community-based continuity of care. These approaches may be especially valuable for high-risk patients, including those with depression, malnutrition, severe dyspnea, low BMI, prior exacerbations, or impaired oxygenation. In these populations, telehealth-supported follow-up, digital symptom monitoring, and remote assessment of clinical deterioration may facilitate earlier intervention, strengthen self-management, and reduce the risk of recurrent hospitalization (86, 87). For patients with persistent hypoxemia or chronic hypercapnia, continuation of LTOT and home-based NIV should also be considered where clinically indicated.

In summary, this study shows that readmission among patients with COPD remains common and increases progressively during the first year after discharge. This substantial burden highlights the need for continuous post-discharge care and early identification of high-risk patients. Several clinical factors, including sex, comorbidity burden, disease severity, nutritional status, psychological status, and need for respiratory support, were associated with readmission risk. These findings provide evidence to support risk-stratified management and individualized interventions aimed at reducing recurrent hospitalization and improving long-term outcomes in patients with COPD.

## Conclusion

This review summarized readmission rates and factors associated with readmission among patients with COPD. The findings showed that readmission remains common during the first year after discharge and is influenced by multiple clinical factors. These results highlight the importance of early identification of patients at high risk of readmission and the implementation of targeted management strategies to improve long-term COPD care. Sensitivity analyses supported the stability of the findings, and the overall methodological quality of the included studies was moderate to high, supporting the credibility and clinical relevance of the conclusions.

### Strengths and limitations

This review has several strengths. It included a large number of studies with a substantial overall sample size, assessed readmission rates at multiple post-discharge time points, and systematically summarized a broad range of factors associated with readmission. The comprehensive search across multiple databases, NOS-based quality assessment, and sensitivity and subgroup analyses strengthened the robustness of the findings. Nevertheless, several limitations should be acknowledged. First, substantial heterogeneity was observed in several pooled analyses, which may be related to differences in study populations, healthcare systems, COPD severity, follow-up duration, readmission definitions, and covariate adjustment across studies. In addition, even after subgroup analyses, several pooled estimates retained wide confidence intervals and substantial heterogeneity, which may limit the precision and generalizability of these findings.

## Data Availability

The original contributions presented in the study are included in the article/Supplementary material, further inquiries can be directed to the corresponding authors.
