# Supplementary material for: Risk factors for hospital readmission in chronic obstructive pulmonary disease: a systematic review and meta-analysis
Source: Front Med (Lausanne). 2026 Jul 14;13:1836031. doi: 10.3389/fmed.2026.1836031 (PMC13408023; doi:10.3389/fmed.2026.1836031)
Supplement: Supplementary file 2 [file Table_2.DOCX]

**Appendix 2.** **Detailed Search Strategy**

**Pubmed**

#1 Pulmonary Disease, Chronic Obstructive [Mesh]

#2 COPD[tiab]

#3 chronic obstructive pulmonary disease [tiab]

#4 chronic obstructive lung disease[tiab]

#5 chronic obstructive airway disease[tiab]

#6 chronic airflow obstruction[tiab]

#7 #1 OR #2 OR #3 OR #4 OR #5 OR #6

#8 Patient Readmission[Mesh]

#9 readmission*[tiab]

#10 rehospitalization[tiab]

#11 rehospitalisation[tiab]

#12 re-hospitalization[tiab]

#13 re-hospitalisation[tiab]

#14 repeat hospitalization[tiab]

#15 repeat hospitalisation[tiab]

#16 multiple admission*[tiab]

#17 recurrent hospitalization[tiab]

#18 recurrent hospitalisation[tiab]

#19 #8 OR #9 OR #10 OR #11 OR #12 OR #13 OR #14 OR #15 OR #16 OR #17 OR #18

#20 Risk Factors[Mesh]

#21 risk factor*[tiab]

#22 predictor*[tiab]

#23 determinant*[tiab]

#24 associated factor*[tiab]

#25 influencing factor*[tiab]

#26 correlate*[tiab]

#27 recurrence[tiab]

#28 protective factor*[tiab]

#29 predictive[tiab]

#30 #20 OR #21 OR #22 OR #23 OR #24 OR #25 OR #26 OR #27 OR #28 OR #29

#31 #7 AND #19 AND #30

**Web of science**

TS=(chronic obstructive pulmonary disease OR chronic obstructive lung disease OR chronic obstructive airway disease OR chronic airflow obstruction OR COPD) AND TS=(readmission* OR rehospitalization OR rehospitalisation OR re-hospitalization OR re-hospitalisation OR repeat hospitalization OR multiple admission*) AND TS=(risk factor* OR predictor* OR determinant* OR associated factor* OR recurrence OR protective factor*)

CNKI

主题 = (慢性阻塞性肺疾病 OR 慢阻肺 OR COPD) AND 主题 = (再入院 OR 再住院 OR 再次住院 OR 重新住院 OR 重返住院) AND 主题 = (危险因素 OR 影响因素 OR 相关因素 OR 预测因素 OR 保护因素)
