# Supplementary material for: Risk factors for hospital readmission in chronic obstructive pulmonary disease: a systematic review and meta-analysis
Source: Front Med (Lausanne). 2026 Jul 14;13:1836031. doi: 10.3389/fmed.2026.1836031 (PMC13408023; doi:10.3389/fmed.2026.1836031)
Supplement: Supplementary file 3 [file Table_3.DOCX]

| **Study** | **Sex(male/female )** | **Country** | **LOS** | **Age** | **Study type** | **Data source** | **Follow-up time point and readmission, n/N (%)** | **Readmission factor** | **Chronic diseases(%)** | **CCI** | **NOS** |
| --- | --- | --- | --- | --- | --- | --- | --- | --- | --- | --- | --- |
| Chan2011 | 50,456/15,041 | China ,Hongkong | / | 76.81±9.59 | POS | HB | 1yr:15,882/65,497 (24.2%) | 1,2,3,7,22 | / | 1.20±0.82 | 6 |
| Lin2013 | 544/148 | China ,  Zhejiang | 16.92±8.76d | 72.95±8.399 | POS | HB | 1m: 49/692 (7.1%) | 5,7 | PVD(33),T2D(59),HTN(29.3),CCP(33.0),IHD(4.8) | / | 6 |
| Peter A.2011 | 44/35 | Manchester, UK | / | 65.3±9.9 | RCS | HB | 3m: 26/79 (32.9%); 1yr: 60/79 (75.9%) | 1,12,13,14,21, | / | 5.48±3.14 | 7 |
| Quintana,J.M.2014 | 2,125/207 | Spain | 6±1.25 | 72.51±9.64 | RCS | HB | 2m:430/2,336 (18.4%) | 5,7,11,12,15,17,25,26 | CHF(77.1) | ≤2:69.25%/>2:(30.75%) | 7 |
| Almagro,2006 | 120/9 | Tarragona, Spain | 12.7±7.3 | 72±9.2 | RCS | HB | 1m: 21/129 (16.3%); 3m: 45/129(34.9%);6m: 53/129 (41.1%); 1yr: 75/129 (58.1%) | / | / | / | 7 |
| Surya .2008 | 43/57 | USA | 14 | 71.9±10.9 | RCS | HB | 1m: 25/100 (25.0%); 3m: 43/100(43.0%); 6m:63/100 (63.0%); 1yr: 23/100(23.0%) | 1,12,13,14,15,21,22, | / | / | 7 |
| Sylvia.2016 | 10,865/5,151 | UK, London | / | 70.8±10.8 | POS | HB | 3m:5,337/15,191 (35.1%) | 1,4,7,15,18,21, | PVD(40.5),TD(19.9) ,pneumonia (20.8) | / | 6 |
| G.Gudmundsson2005 | 199/207 | Norway, Bergen City | 8(6-13) | 69.2±10.5 | CS | HB | 1ry:246/406 (60.6%) | 365:8,13,14,15,22 | CVD(44.6),T2D(10.3) | / | 5 |
| Epstein,D.2018 | 325/214 | Israel, Haifa | 6.99±14 | 69.19±11.75 | RCS | HB | 2m: 59/539 (10.95%) | 1,5,7,9,11,14,21 | HTN(61.6),T2D(33.8),CHF(21.2) | 6.42±3.47 | 6 |
| Sean D 2015 | 1m:69,472/71,509  3m:60,297/63,248 | USA, Pennsylvania | 30:4.30±9.19/4.24±9.05 | 60.1±11.2 | POS | Managing healthcare claim data in the USA | 1m:9,927/123,545 (8.03%); 3m:14,567/123,545(11.79%) | 1,7,11,21 | asthma(27),CRF(9.8),IHD(37.5), lung cancer (6.18), pneumonia (40.4) | 1(15.83)/2(17.35)≥3(57.76) | 8 |
| Roozbeh.2014 | 3,401/4,862 | USA, Texas | 5.1±14.9 | 56.55±5.73 | RCS | Claim data of members of the US healthcare plan | 1m: 741/8,263 (8.9%) | 1,6,7,13,21,25, | 11CCI | / | 7 |
| Mónica2016 | 318/60 | Greece, Athens | 9±1.85 | 71.4±10 | POS | HB | 1m: 68/378 (18.0%) | 5,9,10,16,18 | IHD(7),diabetes 24) | 2±1.48 | 6 |
| Tina.2015 | 117/8 | Spain, Valencia | 8±1.75/7±1.25 | 69.2±9.8 | RCS | HB | 1m:947,084/26,798,404 (3.5%) | 5,7,9,16,20 | CHF(21.6),CRF(0.8),T2D(13.6),NAFLD(2.4),ND(6.4),cancer(9.6%) | / | 6 |
| Christopher.M.2011 | 4,906/4,810 | UK, London | 5days | 73±10 | POS | HB | 3m:2,937/8,586 (34.0%) | 6,7 | 14 comorbidities | 1m:3.94±3.20/3m:3.77±3.07 | 6 |
| M.S.N.Tsui,2016 | 226/24 | China, Hong Kong | ≥4 in 1 year | 76.7±7.7 | POS | HB | 1yr: 183/250 (73.2%) | 1,4,7,8,12,14,15,17,19,23,27,28 | / | 1±0.74 | 6 |
| Chidiamara.2022 | 1222/1226 | Australia | 3.9±3.48 | 72±11.85 | POS | Administrative data of the major public hospitals in Tasmania, Australia | 1m:151/2,261 (6.7%); 3m: 270/2,206 (12.2%);365 d:477/2,009 (23.7%) | 1,21,26 | pneumonia (10.5), Throat and chest pain (5.7)HF(4.6) | / | 6 |
| Alessandra.2020 | 1152/10  59 | Italy, Padua | 9.59±5.80 | 65–84y:1378/Over84y:833 | RCS | Administrative data collected in the Veneto region | 1m:2,211/1,4869 (14.9%) | 7 | 9122 people have at least one comorbid condition |  | 6 |
| Jaber S.2021 | 40/42 | UK | 7±4.68 | 71±10.4 | RCS | HB | 1m: 31/82 (38.0%); 3m: 46/82 (56.0%) | 10,11,13,15,27 | HTN(39),HDL(13),AF(23),HF(16),CKD(16), | 4.3±1.6 | 7 |
| Sara.2020 | 195/58 | Spain, Madrid | 1.3±1.6 | 68.9±9.8y | RCS | HB | 1m: 51/253 (20.2%); 3m: 97/253(56.1 %);1yr:156/253(61.7%) | 16,17,24 | CVD(35.6),T2D(22.5), anemia (25.7) | 1.8±0.9 | 7 |
| Tadahiro.2018 | 30,987/45,710 | USA | / | 76±3 | RCS | HB | 1m:166,668/845,465 (19.7%) | 1,2,3,7,10,21 | / | / | 6 |
| Tadahiro.2017 | 350,967/494,498 | USA | / | 69±0.01 | RCS | Health Insurance and Medical Assistance Service Center | 1m:14,090/76,697 (18.4%) | 1,2,3,7,10,21 | / | 0-1(29)/ 2-3(45)/ ＞4(26) | 6 |
| ChristineL.2013 | 1,031/1,495 | USA | / | 57.75±5.20 | RCS | Truven Health Marketscan® Business Compensation Database | 1m:340/6,095 (5.58%); 3m: 719/6,095 (11.8%); 1yr: 1,681/6,095 (27.58%) | 7,15,19,26 | The 17 comorbidities include 7 cases of lung diseases. | / | 7 |
| Sana.M.H.2017 | 79/2 | Malaysia | 6±3.7 | 72±8.59 | POS | HB | 3m: 33/81 (40.74%) | 7,8,10,12,13,17 | HTN(49.38),diabetes(25.93),IHD(18.52),HDL(17.28),TI(17.28) | 5±1.48 | 5±1.48 |
| Zhou2021 | 332/85 | China, Guangdong | 11±8 | 75±12 | POS | HB | 3m:62/417 (14.9%) | 5,7,21 | HTN(44.4),CPD(2.4),CHF(15.3) | / | 6 |
| Chang2014 | 119/16 | China, Guangdong | / | 66±10.37 | POS | HB | 1yr: 71/135 (52.6%) | 7,12,14,17,21,23,25,27 | HTN(30.4),IHD(20.7),T2D(14.8),CHF(11.1),CVD (4.4) | / | 6 |
| Zhou2022 | 32/111 | China, Henan | / | 73.08±6.44/67.25±5.81 | POS | HB | 1m:42/286(14.7%) | 7,9,27 | T2D(29.51),CHD(52.38),HTN(33.33) | / | 7 |
| David M.2018 | 437,812/618,018 | USA, New York | 2.9±0.78 | 68±4.75 | RCS | National Readmission Database | 1m:202,300/1,055,830 (19.2%) | 1,2,3,7,11,21 | bronchiectasis (27), pneumonia(7.64),asthma(6.97),CHF(5.80),CA(2.44),RF(1.52),MI(1.12) | 5~＞9 | 7 |
| Xiao2024 | 168/112 | China, Sichuan | / | 74.15±6.29/72.97±6.82 | POS | HB | 3m: 92/280 (32.86%) | 1,8,12,13,21 | / | / | 6 |
| Zhang2021 | 175/40 | China, Beijing | / | 74±9.63/75±11.85 | POS | HB | 6m: 55/215 (25.6%) | 1,14,17,22,24 | / | / | 5 |
| Huang2024 | 156/92 | China, Jiangsu | / | 73.61±6.47/71.68±5.95 | POS | HB | 3m: 60/248 (24.2%) | 7,14,23 | CHD(18.3),HTN(48.3),T2D (23.3) | / | 5 |
| Wan2023 | 93/28 | China, Jiangxi | / | ≥75:58/＜75:63 | RCS | HB | 6m: 31/121 (25.62%) | 7,15,16,24 | HTN(32.2),TD(34.7) | / | 7 |
| GuoXJ2024 | 61/39 | China, Hebei | / | 67.54±9.23/67.63±9.25 | POS | HB | 1m: 31/100 (31.00%) | 9,14,17,19,27 | / | / | 5 |
| Lv2015 | 51/27 | China, Beijing | / | 78 | RCS | HB | 1yr: 40/78 (51.28%) | 9,12,17,23 | TD (20.5) | / | 5 |
| Yang2022 | 199/86 | China, Shanghai | 13.53±5.71/10.85±3.77 | 71.47±9.16/70.99±8.264 | RCS | HB | 1yr: 96/285 (33.68%) | 7,24,30 | RF(33.7),CCP(29.8),HTN(13.3),CHF(2.1),TD (9.4) | / | 5 |
| Cao2004 | 155/31 | Singapore | / | 50–95y | POS | HB | 1yr: 85/186 (45.7%) | 5,7,8,10,12,13,14,23 | / | 0 (24.7) /1 (31.8) /≥2(43.5) | 6 |
| Cao2005 | 167/29 | China, Shanghai | / | ＞72:91/≤72:105 | POS | HB | 1yr: 95/196 (48.0%) | 1,3,12,13,14,17,19,23 | / | / | 6 |
| Crisafulli,E.2015 | 117/8 | Spain, Valencia | 8±1.75/7±1.25 | 69.2±9.8 | RCS | HB | 1m: 29/125 (23.2%) | 5,7,9,16,20 | CHF(21.6),CRF(0.8),T2D(13.6),NAFLD(2.4),ND(6.4),cancer(9.6%) | / | 6 |
| JAVIER.2016 | 47,076/5,823 | Spain, Madrid | 10.33±9.83 | 74.79±9.63 | RCS | HB | 1m:52,899/301,794(17.53%) | 1,7,18,20,21 | 16 comorbidities | ＞2:(8.72) | 6 |
| Liu2023 | 163/45 | China, Shanghai | / | 72.92±2.82/  71.78±2.79 | RCS | HB | 1yr: 82/208 (39.42%) | 1,14,18,19,21 | HTN(57.7),T2D(18.3),CHD(18.3) | / | 7 |
| A.Zapatero2013 | 200,841/83,456 | USA | 9.7 | 72.8±15.9 | RCS | HB | 1m:52,428/313,233 (16.7%) | 1,7,11,14,20,21 | HTN (30.6), HF (16.7), T2D (21.7), cancer (12.8), dementia (5.7) | / | 7 |
| C.Fuhrmana2016 | 33,633/21,382 | USA | 8±2 | 72.6±12.9 | RCS | HB | 1m:4,186/58,144 (7.2%); 3m:8,663/58,144 (14.9%); 1yr:18,082/58,144 (31.1%) | 1,10,11,15,21 | / | 1-4 or more | 6 |
| Raquel.2012 | 48,789/226,723 | Spain, Madrid | 11.0±9.8/9.4±8.2 | 72.7±13.7/71.9±15.7 | RCS | HB | 43,067 / 275,521 (15.6%) | 1,7,11,20,21 | HF(20.5),AF(20.52),T2D(27.9),cancer(12.1),CRF(5.93),dementia (5.6) | / | 7 |
| Zhang2014 | 106/31 | China, Beijing | / | ≥75:83/54 | RCS | HB | 1yr: 32/137 (23.4%) | 1,5,9,13,15,17 | CHF(12.4), cardiac inadequacy (4.4), stroke(8.6),T2D(20.4),PVD(36.5) | / | 6 |
| Andrea S.2019 | 71,323/54,689 | UK | 12±10.37 | 35y-84y | RCS | Managing healthcare claim data in the USA | 1m:49,046/252,756 (19.4%) | 1,3,7,10,11,21,26 | Asthma(24.7),CHF(21.4),T2D(13.8),diabetes(10.4),RF(7.8),KD(6.6),AMI(5.2),cancer(4.9),dementia (4.2),PVD(2.5),CVD(2.1) | / | 6 |
| Li2022 | 16,771/4,036 | Beijing, China | 17.8±12.2 | 74.7±10.7 | RCS | Beijing Hospital Database | 1m:20,808/131,591 (15.8%) | 1,4,7,11,15 | HTN(57.5),T2D(21.2),osteoporosis(4.2),CHD(49.6),CVD(27.1),CHF(16.4), anxiety-depression (92.5),cancer(4.3) | 0:30.4/1:29.2/2:19.7/＞3:20.7 | 7 |
| Zhou2020 | 58/228 | China, Guangdong | / | 69.3±10.1/68.6±11.1 | RCS | HB | 1m: 42/286 (14.68%) | 1,9,7,14,21,27 | CHD(33.2),HTN(32.9),IHD(5.2),T2D(33.2) | / | 6 |
| Chen2020 | 60/32 | China, Guangdong | ＜2w：36/≥2:56 | 72.19 ± 8.45 | RCS | HB | 1m: 46/92 (50.0%) | 4,7 | / | / | 5 |
| Chen2024 | 226/15 | China, Zhejiang | / | ＜65(19.1),65~75(51.0),＞75(29.5) | POS | HB | 1m: 33/241 (13.69%) | 7,19 | CHD(48.1),HTN(60.0),IHD(5.2),T2D(19.1),PHD(48.1) | / | 5 |
| Ding2022 | 232/98 | China, Anhui | 11.85±3.88/10.96±3.47 | 67.41±16.98/65.02±16.60 | RCS | HB | 1m: 53/330 (16.1%) | 13,30,31 | CHD(22.4),HTN(25.8),T2D(17.9) | / | 6 |
| Feng2022 | 365/131 | China, Sichuan | 13±7/15±10 | 74±13/73±14 | CS | HB | 3m: 112/496 (22.6%) | 9,12,19,22,23,30 | bronchiectasis (17.3),T2D(16.7),CHD(6.9),pulmonary interstitial fibrous (4.6) | / | 5 |
| Zhang2023 | 124/86 | China, Hubei | 8.9±1.8/9.0±1.9 | 68.1±8.9/68.2±8.8 | CS | HB | 1m: 33/241 (13.7%) | 9,23,27, | HTN(25.0),T2D(12.5)/CHD(20.0) | / | 5 |
| Ma2024 | 80/32 | China, Anhui | 9.12±1.33/8.85±1.46 | 69.0±3.2/68.0±4.2 | CS | HB | 1m: 34/122 (27.9%) | 9,12 | HTN(23.5),T2D(14.7),CHD(17.6) | / | 5 |
| Sigrun A.2013 | 1422/1754 | USA | 6±1.5 | 71.1±9.11 | POS | Danish healthcare database | 1ry: 663/3,176 (20.9%) | 1,7,14,16,22 | 28 commodities | / | 7 |
| GuoZQ2024 | 91/77 | China, Hebei | 11.46±3.04/10.94±2.57 | 70.11±14.38/68.61±9.38 | POS | HB | 1m: 38/168 (22.6%) | 9 | HTN(48.46),T2D(24.62),CHD(14.62) | / | 5 |

Abbreviation: Retrospective Cohort Study: RCS; Prospective Observational Study: POS; CH: Cohort study; Neurological diseases: ND; Congestive Heart-Failure: CHF; Tuberculosis Infection: TI; Ischemic Heart Disease: IHD; Type 2 diabetes: T2D; Hypertension: HTN; Chronic Cor Pulmonale: CCP; Cardiac Arrhythmia: CA; Atrial Fibrillation: AF; Chronic Kidney Disease: CKD; Hyperlipidemia: HDL; Obstructive Sleep Apnea Syndrome: OSAS; Respiratory Failure: RF; Acute Myocardial Infarction: AMI; Peripheral Vascular Disease: PVD; Hospital Base: HB

; NAFLD: Non-cirrhotic liver disease

Risk factor: 1. Gender; 2. Fee payment method; 3. Discharge destination (care environment); 4. Charlson Comorbidity Index (CCI); 5. Arterial blood oxygen level (PaO₂); 6. Alcohol history; 7. Comorbid chronic diseases (such as hypertension, diabetes, cardiovascular disease); 8. Anxiety; 9. Inflammatory markers; 10. History of previous COPD exacerbations; 11. Length of stay (LOS, days); 12. Lung function level; 13. Depression; 14. Smoking history; 15. Number of hospitalizations in the past year; 16. Degree of dyspnea; 17. modified Medical Research Council (mMRC score); 18. Ventilatory Support; 19. Drug Treatment Situation; 20. Malnutrition Status; 21. Age; 22. B-type natriuretic peptide (BNP) level; 23. Body Mass Index (BMI); 24. Race; 25. History of long-term oxygen therapy; 26. Number of previous emergency department visits; 27. COPD Assessment Test (CAT); 28.Six minutes’ walk distance (6MWD)

**Supplementary Table 2. Meta-analysis of risk factors for all-cause COPD readmission within one month**

| **Risk factors of readmission within 30 days** | **Studies** | **Heterogeneity test** | | **OR (95% CI)** | ***p*** |
| --- | --- | --- | --- | --- | --- |
|  |  | ***I^2^*** | ***P*** |  |  |
| **Gender** |  |  |  |  |  |
| male | 11 | *99.4%* | 0.0027 | 1.2522 [1.0809; 1.4506] | 0.000 |
| Female | 5 | *99.0%* | < 0.0001 | 0.9314 [0.8750; 0.9913] | 0.0255 |
| **Age** |  |  |  |  |  |
| 45~65 | 7 | *98.2%* | < 0.0001 | 1.2463 [0.9912; 1.5672] | 0.0595 |
| 66~75 | 6 | *70.0%* | 0.0052 | 1.0137 [0.9750; 1.0540] | 0.4929 |
| 75~85 | 5 | *85.4%* | < 0.0001 | 1.3392 [0.8559; 2.0956] | 0.2010 |
| ≥85 | 3 | *48.6%* | 0.1426 | 0.9791 [0.9342; 1.0262] | 0.3780 |
|  |  |  |  |  |  |
| **oxygenation** |  |  |  |  |  |
| PaO2 | 2 | 0 | *0.5610* | 1.0222[1.0093;1.0353] | 0.0007 |
| PaO2/FiO2 | 1 | / | */* | 0.9800[0.9701;0.9901] | *P ＜0.001* |
| **laboratory indicators** |  |  |  |  |  |
| WBC | 2 | 56.8% | *0.1282* | 1.9596[0.4493;8.5465] | *0.3706* |
| BNP | 1 | / | */* | 0.9900 [0.9796; 1.0006] | *0.0631* |
| RDW | 2 | 0 | *0.4920* | 2.0703 [1.3203; 3.2464] | *0.0015* |
| NLR | 2 | 87.9% | *0.0040* | 2.7184 [0.2482; 29.7704] | *0.4128* |
| CRP | 5 | 28.8% | *0.2292* | 1.8458 [1.4972; 2.2755] | *< 0.0001* |
| **CCI** |  |  |  |  |  |
| 1 | 3 | 96.8% | < 0.0001 | 1.0679 [0.9231; 1.2354] | 0.3767 |
| 2 | 8 | 98.9% | < 0.0001 | 1.1542 [1.0533; 1.2649] | 0.0021 |
| ≥3 | 5 | 97.0% | < 0.0001 | 1.5215 [1.4905; 1.5531] | < 0.0001 |
| ≥ 5 | 1 | / | / | 2.2500 [1.6627; 3.0448] | < 0.0001 |
| **Chronic disease** |  |  |  |  |  |
| atrial fibrillation | 2 | 98.9% | < 0.0001 | 1.0675 [0.8678; 1.3132] | 0.5365 |
| anemia | 2 | 25.1% | 0.2479 | 1.2500 [1.2131; 1.2881] | < 0.0001 |
| asthma | 3 | 98.3% | < 0.0001 | 0.8666 [0.6157; 1.2198] | 0.4118 |
| cancer | 6 | 97.3% | < 0.0001 | 1.2892 [1.0763; 1.5444] | 0.0058 |
| coronary artery disease | 3 | 71.2% | 0.0310 | 1.8994 [0.8153; 4.4247] | 0.1371 |
| diabetes | 9 | 82.1% | < 0.0001 | 1.0199 [0.9741; 1.0679] | 0.4003 |
| Hypertension | 4 | 0 | 0.4930 | 0.9905 [0.9653; 1.0163] | 0.4661 |
| ischemic heart disease | 3 | 55.4% | 0.1064 | 1.0349 [0.9816; 1.0911] | 0.2036 |
| obesity | 3 | 82.0% | 0.0039 | 0.8503 [0.8122; 0.8902] | < 0.0001 |
| Congestive Heart Failure | 6 | 96.6% | < 0.0001 | 1.1154 [1.0017; 1.2421] | 0.0464 |
| dementia | 2 | 0 | 0.4607 | 1.0003 [0.9636; 1.0384] | 0.9867 |
| renal failure | 2 | 93.8% | < 0.0001 | 1.1211 [0.9918; 1.2672] | 0.0674 |
| cerebrovascular disease | 2 | 93.0% | 0.0002 | 0.9378 [0.7450; 1.1806] | 0.5848 |
| osteoporosis | 2 | 28.9% | 0.2356 | 1.1788 [1.0724; 1.2958] | 0.0047 |
| **Alcohol** | 2 | 0 | 0.7773 | 1.1292 [1.0950; 1.1643] | < 0.0001 |
| **History of acute attacks** | 2 | 92.4% | 0.0003 | 1.5119 [0.6200; 3.6869] | 0.3634 |
| **hospitalization days** |  |  |  |  |  |
| ≤7 | 8 | 99.2% | < 0.0001 | 1.1892 [1.0868; 1.3013] | 0.0002 |
| ＞7 | 10 | 99.7% | < 0.0001 | 1.4420 [1.0988; 1.8925] | 0.0083 |
| **lung function** |  |  |  |  |  |
| FEV1% < 50% | 4 | 77.8% | 0.0037 | 1.1986 [0.6782; 2.1183] | 0.5329 |
| **ventilatory support** | 4 | 94.9% | < 0.0001 | 1.2736 [1.0460; 1.5506] | 0.0160 |
| **drug therapy** | 3 | 95.4% | < 0.0001 | 1.3854 [0.6156; 3.1178] | 0.4309 |
| **malnutrition** | 5 | 92.1% | < 0.0001 | 1.4375 [1.2039; 1.7165] | < 0.0001 |
| **Long-term oxygen therapy** | 2 | 96.5% | < 0.0001 | 1.0177 [0.9405; 1.1013] | 0.6627 |
| **Previous emergency treatment** | 8 | 99.3% | < 0.0001 | 1.2653 [1.0603; 1.5098] | 0.0091 |
| **Depression** | 2 | 0 | 0.6307 | 1.0955 [1.0410; 1.1530] | 0.0005 |

**Supplementary Table 3. Meta-analysis of risk factors for COPD readmission within three months**

| **Risk factors of readmission within 90 days** | **Studies** | **Heterogeneity test** | | **OR (95% CI)** | ***p*** |
| --- | --- | --- | --- | --- | --- |
|  |  | ***I^2^*** | ***P*** |  |  |
| **Gender** |  |  |  |  |  |
| male | 11 | 0.0027 | *99.4%* | 1.2522 [1.0809; 1.4506] | 0.000 |
| Female | 5 | < 0.0001 | *99.0%* | 0.9314 [0.8750; 0.9913] | 0.0255 |
| **Age** |  |  |  |  |  |
| 66~75 | 15 | < 0.0001 | *99.8%* | 0.269 [0.205; 0.344] | < 0.0001 |
| **pattern of payment** |  |  |  |  |  |
| public assistance | 6 | < 0.0001 | *99.6%* | 1.1581 [1.0290; 1.3034] | 0.0150 |
| private insurance | 2 | < 0.0001 | *99.6%* | 0.7513 [0.4382; 1.2880] | 0.2985 |
| self-paying | 2 | < 0.0001 | *98.2%* | 0.8493 [0.6013; 1.1996] | 0.3538 |
| **Discharge destination** |  |  |  |  |  |
| Home medical care | 3 | p < 0.0001 | *94.2%* | 1.2847 [1.2024; 1.3727] | < 0.0001 |
| Professional nursing institution | 4 | p < 0.0001 | *99.2%* | 1.1995 [0.9672; 1.4876] | 0.0977 |
| Disobeying medical advice | 2 | p = 0.0669 | *70.2%* | 1.9739 [1.7448; 2.2332] | < 0.0001 |
| **CCI** |  |  |  |  |  |
| 1 | 1 |  |  | 1.0650 [0.9811; 1.1561] | 0.1326 |
| 2 | 2 | p = 0.4628 | *0* | 1.1052 [1.0409; 1.1734] | 0.0011 |
| ≥3 | 1 | / | */* | 1.3000 [1.2066; 1.4006] | < 0.0001 |
| **laboratory indicators** |  |  |  |  |  |
| PaO2 | 1 | / | */* | 1.0300 [0.9529; 1.1134] | 0.4566 |
| Lung function |  |  |  |  |  |
| FEV1% < 50% | 2 | 0.0011 | *90.6%* | 0.5998 [0.0511; 7.0381] | 0.6841 |
| **ventilatory support** | 2 | 0.7625 | *0* | 1.1298 [1.0351; 1.2331] | 0.0063 |
| **Chronic disease** |  |  |  |  |  |
| diabetes | 3 | 87.6% | 0.0003 | 1.6258 [0.5031; 5.2545] | 0.4168 |
| ischemic heart disease | 3 | 92.9% | < 0.0001 | 1.1256 [0.9230; 1.3725] | 0.2426 |
| Stroke | 2 | 50.7% | 0.1545 | 1.0069 [0.9137; 1.1097] | 0.8889 |
| Cancer | 2 | 88.2% | 0.0036 | 1.1075 [0.8295; 1.4785] | 0.4888 |

**Supplementary Table 4. Meta-analysis of risk factors for COPD readmission within one year**

| **Risk factors of readmission within 365 days** | **Studies** | **Heterogeneity test** | | **OR (95% CI)** | ***p*** |
| --- | --- | --- | --- | --- | --- |
|  |  | ***I^2^*** | ***P*** |  |  |
| **Gender** |  |  |  |  |  |
| male | 3 | 68.0% | *0.0440* | 1.5514[0.8085; 2.9768] | 0.1866 |
| Female | 2 | 0 | *0.1348* | 1.1047[0.9695; 1.2587] | 0.5301 |
| **Aage** |  |  |  |  |  |
| 66~75 | 5 | 67.5% | *0.0153* | 1.0429 [0.9827; 1.1069] | 0.1663 |
| PaO2 | 2 | 85.6% | *0.0084* | 1.7668 [0.2826; 11.0471] | 0.5428 |
| **Lung function** |  |  |  |  |  |
| FEV1% < 50% | 7 | 75.2% | *0.0005* | 1.3637 [1.0255; 1.8135] | 0.0329 |
| **Chronic disease** |  |  |  |  |  |
| Hypertension | 3 | 65.2% | *0.0563* | 1.3944 [0.8814; 2.2059] | 0.1554 |
| diabetes | 2 | 65.0% | *0.0908* | 1.6111 [0.7659; 3.3887] | 0.2087 |
| **breathing difficulties** | 4 | 41.0% | *0.1659* | 2.0209 [1.4983; 2.7259] | < 0.0001 |
| MRC≥3 | 1 | / | */* | 1.8900 [0.8999; 3.9694] | 0.0927 |
| **drug therapy** | 2 | 76.5% | *0.0390* | 3.1967 [0.3438; 29.7198] | 0.3070 |
| **Depression** | 4 | 0 | *0.6028* | 1.3701 [1.1387; 1.6486] | 0.0009 |
| **BMI(＜18.5)** | 4 | 63.4% | *0.0422* | 3.0142 [1.6921; 5.3695] | 0.0002 |
| **Race** |  |  |  |  |  |
| Caucasian | 2 | 92.3% | *0.0003* | 1.0322 [0.9349; 1.1395] | 0.5308 |
| Spanish nationality | 2 | 83.2% | *0.0148* | 0.9385 [0.8593; 1.0250] | 0.1582 |
| Black | 2 | 98.3% | *< 0.0001* | 1.0177 [0.9405; 1.1013] | 0.6627 |
| **anxiety** | 2 | 84.4% | *0.0113* | 1.8817 [0.5217; 6.7880] | 0.3341 |
| Inflammatory |  |  |  |  |  |
| CRP | 2 | 58.9% | *0.1188* | 1.3403 [0.9809; 1.8315] | 0.0660 |
| smoking history | 5 | 96.7% | *< 0.0001* | 0.9846 [0.4484; 2.1617] | 0.9691 |
